# Medical Ethics in Plastic Surgery: A Mini Review

**Published:** 2016-09

**Authors:** Nasrin Nejadsarvari, Ali Ebrahimi, Azin Ebrahimi, Haleh Hashem-Zade

**Affiliations:** 1 Deputy of Research, Tehran University of Medical Sciences, Tehran, Iran;; 2 Department of Plastic Surgery, Baqiyatallah University of Medical Sciences, Tehran, Iran;; 3 Tehran University of Medical Sciences, Tehran, Iran;; 4 Dental Research Center, Tehran University of Medical Sciences, Tehran, Iran

**Keywords:** Medical ethics, Plastic surgery, Aesthetic

## Abstract

Currently, cosmetic surgery is spread around the world. Several factors are involved in this rapidly evolving field such as socio-economic development, changes in cultural norms, globalization and the effects of Western culture, advertising, media, and mental disorders. Nowadays the cosmetic surgery is becoming a profitable business, which deals exclusively with human appearance and less from the perspective of beauty based on physical protests and considering factors such as sex, age, and race. The morality of plastic surgery subspecialty has undergone many moral dilemmas in the past few years. The role of the patient regardless of his unrealistic dreams has questionable ethical dimension. The problem is the loss of human values and replacing them with false values, of pride and glory to a charismatic person of higher status, that may underlie some of the posed ethical dilemmas. Cosmetic surgery has huge difference with the general principle of legal liability in professional orientation, because the objective for cosmetic surgeries is different from common therapeutic purposes. To observe excellence in the medical profession, we should always keep in mind that these service providers, often as a therapist (healer) must maintain a commitment and priority for patient safety and prior to any action, a real apply for this service recipient should be present. Also, patient–physician confidentiality is the cornerstone of medical ethics. In this review, we study the issues addressed and the ways that they can be resolved.

## INTRODUCTION

Today, cosmetic surgery is growing and there are several factors that are involved in this rapid development. Factors such as economic development, social and cultural norms changes, globalization and exposure to culture media and repeated trips to the Western countries are the main causes for this rapid development. Cultural changes and developments in different societies have increased concerns over their appearance and subsequently, these increase the demands for plastic surgeries by people.^1^ But, despite all these factors, the demand for cosmetic surgery is generally motivated by psychosocial factors. The desire for beauty in human nature has long existed since the creation of the features. They have interest to modify and improve their appearance and surroundings, respectively.^2^ Consistent with the idea that woman are under greater pressure than men to attain current ideals of beauty and thinness, more women than men usually express an interest in cosmetic procedures, while this interest is an appearance orientation.^3^

Therefore, factors which influence the consumers today worry people to their physical appearance or body image. Therefore, the extent that the community is exposed to advertisings in the media for new models, it would have more raised ethical questions.^4^ Traditionally the cosmetic surgeries performed by plastic surgeons due to a high demand and to obtain more income, the profit aspects of the business are highlighted. Therefore, other surgeons in different expertise are also attracted and unfortunately in some areas, aesthetic surgical procedures are performed by this groups.^5^ In light of these trends, the human body, like an inanimate object, material or merchandise is tarnished.^6^ Moreover, exaggerated advertisings and unrealistic media would cheat the community and encourage them to apply for cosmetic surgeries. Today, the importance of this area of expertise has been reduced, its actual position is lost and the plastic surgery is going in the transition from acute ethical problems.^6^

The word “plastic” in plastic surgery, is a Greek word meaning “to form”. Plastic surgery is divided into two sections, reconstructive surgery and cosmetic surgery.^7^ The initial idea of the genesis of plastic surgery is returned to repair the deep wounds of the Neanderthals due to the rock, cool guns, and spears back and bite animals.^7^ Historical records remained in countries like Egypt, India, Greece, Rome, in relation to wound healing; and the use of other body tissues to reconstruct the nose and lip, repair the cleft lip and tumors, correct congenital anomalies, etc. All point to the initial steps in the era of plastic surgery.^7^ Plastic surgery in the nineteenth century progressed and at this time for first time, the skin graft was performed in Milan.^7^ Plastic surgery, including reconstructive and cosmetic surgery, writings on clay in Mesopotamia in ancient Persia showed the wound healing and congenital anomaly repair to be born in that period.^7^

Plastic surgeries were developed between the two world wars. Advertisements in magazines and newspapers in this area lead to the spread of these practices and had a key role in most people’s concerns wished to change their physical appearance. Females are more likely to apply for cosmetic surgery, as they wish to be more attractive at their jobs and to have a higher social status, to seem younger, and due to psychological, social and personality factors.^2^ Social and community factors play important roles as these factors are related to cultural norms changes, globalization and the expansion of the Western culture receiving through the media and travels. In today’s consumer-oriented societies, attention to body image has become very popular, and the media has important role.^8^

In America, one out of every 150 cases performs breast aesthetic surgery and research has shown that it is born of the stars and models in the magazines publishing their photos and creates new norms in the society.^9^ Advances in reconstructive surgery have resulted into a major revolution in the treatment of patients with congenital anomalies, burns, and carcinomas of the skin.^9^ Also, cosmetic surgery has developed to revise and change the appearance in order to improve the confidence.^10^ In recent years, the abuses in medical ethics in plastic surgery have greatly increased, and high ethical questions are raised. Is cosmetic surgery a job (business) or is it necessary as a part of health care system of the patient best interest? Specialized field of plastic surgery has been subject to many ethical dilemmas. The issue is in both reconstructive and cosmetic surgery, even it is more in cosmetic surgery. There are fewer cases of bioethical problems in reconstructive surgery and its popularity in different countries is created the ways to “rebuild the human body”.^11^

The main subject is the lack of agreement on the terminology of cosmetic surgery, therefore, divisions such as cosmetic medicine, cosmetic dermatology, cosmetic surgery in office, have made public disturbances. Todays, there is a trend to less aggressive procedures in academic and private centers.^12^ Indeed, according to some statistics, 65% of all cosmetic procedures are now non-surgical,^13^ leading to a grow in patient’s interest to cosmetic interventions leading to an exponential rise in cash flowing into the market for lasers, fillers and Botox.^14^ Cosmetic surgery or any medical or surgical manipulation performed by physicians with no formal training shows an increasing debated critical issue.^3^ Cosmetic surgery traditionally performed by plastic surgeons are acceptable, but today, other surgeons tend to perform these procedures, and even the non-physicians as well, therefore cosmetic procedures may be performed by non-qualified persons. The lack of academic and practical skills and beauty service providers can be considered in violation of the ethical principle of “Nonmaleficence”.^3^

Advertising and public deception is the ethical problem of plastic surgery. Media forge some surgeons by intensive advertisings of body image, claiming a lie, exaggerating individual capability using photoshop images in the offices, that all provide ethical problems in the field of plastic surgery.^7^ At present, aesthetic surgery has become increasingly popular around the world^3^ and this medical profession has become a profitable business as “cosmetic surgery industry”.^7^ Plastic surgery corrects the defects and pays less attention to the culture and beauty in relation to physical appearance and attention to characteristics such as gender, age, and race.^6^ In this field, health services are offered commercially.^6^

In addition to the desire for change and attractiveness, financial and economic factors are the main factors that play a role in encouraging people for cosmetic surgery, because, some jobs require access to the beautiful appearance and there are a tendency of individuals to perform a cosmetic surgery.^2^ However, in today’s modern society, the elderly people perform cosmetic surgery for rejuvenation. Today, in light of this trend, genuine human values are lost and false values, such as pride and ambition have been replaced. Apart from all these, a large percentage of those seeking cosmetic surgery have psychological factors.^15^ Typical patients are those with body dysmorphic disorders (BDD) that have the preoccupation of a defect in their appearance.^15^

These procedures may impose worse complications to the patient than the plastic surgery procedures.^11^ There are other ethical problems regarding age, especially in adolescent age group that adolescents because of dissatisfaction with their appearance and low self-esteem may experience social isolation. Recently, requests on the youth beauty have been high, and too much attention has been toward appearance changes which are mostly due to false information through the media. The media information, ultimately leads to unrealistic expectations by teens that may have destructive effects on plastic surgery.^11^

In addition to these problems, the field of plastic surgery has common problems such as inability to establish a friendly relationship with the patient, taking informed consent, inability to take medical history, failure to diagnose properly, treatment of complications, and coping false statements, that all these problems may be faced by some experts.^7^ Ethical issues in cosmetic surgery can conflict with the four principles of medical ethics too.^16^ According to the principle of autonomy, there is a need to take informed consent from adult patients freely by a qualified plastic surgeon before any surgery. Therefore, the plastic surgeon must provide sufficient information regarding the procedure, type of surgery, its complications, alternative treatment methods, costs, and the risks of surgery that should be understandable by each patient.^17^ Because plastic surgery procedures in most cases are electively, and accomplished based on patients’ demands, physicians that do not prevent harmful surgeries will be inconsistent with the principle of autonomy of the patient.^17^

Although plastic surgeries designed as elective procedures, they have long-term effects on body function and health,^17^ so the demands and expectations of the patient is very important. This ethical issue in surgical procedures such as massive liposuction with more surgical damages and complications are problematic assumptions. According to the principle of benefit, attention to the interests of the patient must always be considered. But in cosmetic surgery, the patient’s benefits are always a big moral dilemma.^18^ The principle of Non-maleficence, bans any acts against the interests of the patient and therefore, the unrealistic expectations of patients would have ethical problems. So in order to fulfill this principle, the patient expectations should be realistic, must measure the profit and loss of the patient, have to consider the medical condition of the patient, denote to the possibilities of resuscitation for the patient in the surgical center if necessary, and pay attention to the specialized facilities including equipments and personnel necessary for cosmetic surgeries**. **According to the principle of justice, allocating resources should take priority based on clinical needs**. **Because cosmetic surgeries are elective and non-emergent, if we take these procedures in governmental hospitals with a shortage of health care resources, the problem would be an important moral dilemma.^2^

The cosmetic surgeries tend to be justified under various titles,^1^^,^^19^ including definition of health by WHO for the convenience of complete physical, mental, social and spiritual aesthetic surgeries that should be considered a step towards improving the health status of individuals. The care is considered based on the autonomy that an individual wishes to become more beautiful. It would be a mistake to think that, with respect to the body, one can adopt a purely external stance. Body and mind are not two separate areas of human life; and they come together in one person. The ability to cope with both, by harmonizing them, is not simple, straightforward, or self evident. The mind never reigns over the body completely, nor does the body rule the mind.^20^

Unfortunately, the general population believe that cosmetic surgery has lead to such interest; outward beauty, happiness, pride and success, while the real value of people is not only restricted to physical appearance, and attract the policy-makers on health status in the society with the right culture. Legal liability in cosmetic surgery is very different from general principles of professionalism, because the objectives are different from common therapeutic targets. In fact, plastic surgeon does not face with a patient, but is encountered with a healthy person who is seeking beauty and to feel younger, which it is more important to carefully assess the patient’s expectations. On the other hand, the results of the surgery are not always in accordance with the patients’ wishes, so surgeons must get informed consent that explains the risks and fully understand the possible unwanted cosmetic complications.^21^

Plastic surgeons in addition to academic skills require special precision on complications that may be associated with these operations and subsequent complaints of the patients that may take place. Therefore, the surgeon must have a professional responsibility to take any procedure on behalf of his personal experience.^11^ On the other hand, an experienced physician for a patient with suspected BDD requests psychiatric consultation.^22^^,^^23^ The goal of reconstructive surgery is restoring functional disorders resulting from trauma, accidents, diseases and congenital defects.^7^ Hence, there is lower ethical problems in comparison to aesthetic surgeries because non-plastic surgeons do less involvement in these procedures and also the patient’s expectations is lower.^7^

Transplantation in plastic surgery that is not life-sustaining are accomplished to maintain the integrity of the physical appearance and improve the patient’s health by improving the quality of life and psychosocial aspects, and it can be done with consideration of the fit between benefits and risks. The experimental aspects of this surgeries and possible complications with immunosuppressant’s therapy should be considered**, **providing an informed consent from the patient or the other recipient of services based upon the principles of autonomy and privacy. This has become the requirement at the center of morally valid decision making in health care and research.^17^

So at the time of informed consent taking, particular attention must be done if there is the uncertainty of the results, the complexity and the nature of the operation, risks, or post-operative complications.^24^ In reconstructive transplantation surgery, equitable allocation of resources should be raised. Of note that it is always necessary to provide expert advice by a team of physicians, psychiatrists, psychologists, physiotherapists and if necessary, to be continued at the appropriate time and even the families must be involved in plastic surgery transplantation procedures.^11^ There are several ethical dilemmas in the field of plastic surgery, and the scientific community to develop relevant professional codes of ethics-related trend, including the code of ethics by American Society of Plastic Surgeons^25^ that was the first code written in this area. 

Later, the International Society of Plastic Surgeons, have developed a coherent code that renders the preservation of human dignity, of academic and practical skills, the protection of society against incompetent doctors, observing all the rules and regulations of the profession, giving priority to the interests of the patient, and consultation on the issue.^26^ Patient–physician confidentiality is the cornerstone of medical ethics. Medical confidentiality has been among the main principles of medicine for longtime and has been respected by the physicians. If the patient does not trust the physician, he will not go on a visit, or if he does, he will not give adequate description of his condition.^27^

The first factor must deal with ethical dilemmas in plastic surgery. It is an effort to determine the terminology related measures, the scope of practice in this field and different specialists that have qualify for doing these procedures. In addition, the law should act as moral support, and surgeons that have license to practice plastic surgery, must be announced through relevant institutions, such as the Medical Council and Scientific Society of Plastic Surgeons in every country for the public and they must prevent unspecialized doctors to mislead the public with false titles and the purposes of just profit seeking. In this regard, governor and health ministry should help patients if it is necessary.^28^

 Similarly, a legal entity should be written in the advertising and media related to plastic surgery and legal act with physicians that exaggerate the results of their plastic surgery with Photoshop. At the same time, reform of public opinion in relation to cosmetic surgery is necessary to explain the real value of humanity. The human real value is not limited to the changes in the appearance. Attitudes in the society and medicine also have a significant role in the tendency of people. Consultation may be required before any plastic surgery including cosmetic surgery, in this context, to assess the mental and physical condition of the patient prior to any procedures. 

In order to identify the risks and benefits of cosmetic surgery, it is necessary to promote the public education and to correct the society attitude. For any procedures that are done for juveniles, “realistic expectations” should always be considered in all aspects of cosmetic and reconstructive surgery procedures in this age group. It is essential to justify the policies governing the relations between patient and physician in order to dampen the demand and the impact of interventions to achieve the desired objective. It must always be kept in mind that the plastic surgeons, in addition to cosmetic surgery procedures, often are as a healer and moral obligation protects the patient health as a priority. Finally, a reminder that promotes professionalism training in plastic surgery is mandatory, also the ethical aspects must be considered in any plastic surgery and it is necessary to draw special attention to pundits professionalism guidelines required for plastic surgery.

## CONFLICT OF INTEREST

The authors declare no conflict of interest.
